# A New *MAMLD1* Variant in an Infant With Microphallus and Hypospadias With Hormonal Pattern Suggesting Partial Hypogonadotropic Hypogonadism—Case Report

**DOI:** 10.3389/fendo.2022.884107

**Published:** 2022-06-28

**Authors:** Diego Yeste, Cristina Aguilar-Riera, Gennaro Canestrino, Paula Fernández-Alvarez, María Clemente, Núria Camats-Tarruella

**Affiliations:** ^1^Section of Paediatric Endocrinology, Vall d’Hebron University Hospital, Barcelona, Spain; ^2^CIBER of Rare Diseases (CIBERER), Instituto de Salud Carlos III (ISCIII), Madrid, Spain; ^3^Paediatric Endocrinology Service, Paediatric Service, Sant Joan de Déu Manresa Hospital, Manresa, Spain; ^4^Laboratory of Clinical and Molecular Genetics, Vall d’Hebron University Hospital, Barcelona, Spain; ^5^Growth and Development Research Group, Vall d’Hebron Research Institute, Vall d’Hebron University Hospital, Barcelona, Spain

**Keywords:** *MAMLD1* gene, hypospadias, minipuberty, hypogonadotropic hypogonadism, microphallus, different sex development (DSD)

## Abstract

*MAMLD1* (X chromosome) is one of the recognized genes related to different sex development. It is expressed in testis and ovaries and seems to be involved in fetal sex development and in adult reproductive function, including testosterone biosynthesis. However, its exact role remains unclear. Over 40 genetic variants have been described, mainly in male individuals and mostly associated with hypospadias. Although MAMLD1 has been shown to regulate the expression of the steroidogenic pathway, patients with *MAMLD1* variants mostly show normal gonadal function and normal testosterone levels. Here we describe a patient (46,XY) with hypospadias and microphallus, with low testosterone and dihydrotestosterone (DHT) levels, and with inappropriately low values of luteinizing hormone (LH) during minipuberty. This hormonal pattern was suggestive of partial hypogonadotropic hypogonadism. A stimulation test with hCG (4 months) showed no significant increase in both testosterone and dihydrotestosterone concentrations. At 5 months of age, he was treated with intramuscular testosterone, and the penis length increased to 3.5 cm. The treatment was stopped at 6 months of age. Our gonadal function massive-sequencing panel detected a previously unreported nonsense variant in the *MAMLD1* gene (c.1738C>T:p.Gln580Ter), which was classified as pathogenic. This *MAMLD1* variant, predicting a truncated protein, could explain his genital phenotype. His hormonal profile (low testosterone, dihydrotestosterone, and LH concentrations) together with no significant increase of testosterone and DHT plasma concentrations (hCG test) highlight the potential role of this gene in the biosynthesis of testosterone during the fetal stage and minipuberty of the infant. Besides this, the LH values may suggest an involvement of MAMLD1 in the LH axis or a possible oligogenesis. It is the first time that a decrease in DHT has been described in a patient with an abnormal *MAMLD1*.

## Introduction

*MAMLD1* (Xq28, OMIM 300120) is one of the genes related to different sex development (DSD) (1, 2). It is expressed in human fetal and adult testes and in human ovaries (1, 3, 4), and it seems to be involved in sex development in fetal life and in adult reproductive function. It contributes to the development and formation of the male external genitalia in the late stages of fetal organogenesis (weeks 8–12) (3), and it has been related to testosterone biosynthesis in mice (5–7). Follow-up studies in patients with variants in *MAMLD1* diagnosed in infancy have shown an impaired testicular function with aging (8), but patients can be fertile (4). Interestingly, MAMLD1’s exact role is still not clear (4, 9, 10). Recently, it has also been related to early-onset obesity (11).

The variants in *MAMLD1* are inherited in an X-linked recessive fashion. Over 40 *MAMLD1* sequence variations have been described (12) not only mainly in males and mostly associated with hypospadias (1, 4, 12–18) but also together with microphallus (4, 12, 14, 15, 19) and/or cryptorchidism (1, 4, 12, 13, 15). More severe DSD cases such as 46,XY individuals with female external genitalia (1, 4) and 46,XY with complete gonadal dysgenesis (20) have been described. Furthermore, in 46,XX females, one homozygous *MAMLD1* variant was also reported in a patient with gonadal dysgenesis, primary amenorrhea, bilateral streak gonads, and clitoromegaly (21) and in one heterozygous female patient who presented primary ovarian insufficiency (9).

Although MAMLD1 has been shown to regulate the expression of the steroidogenic pathway, patients with *MAMLD1* variants mostly show normal gonadal function and normal testosterone levels (12). Here we describe a patient with hypospadias and microphallus, with low testosterone and dihydrotestosterone (DHT) levels, and with inappropriately low values of luteinizing hormone (LH) due to a variant in the *MAMLD1* gene.

## Background

The patient is a 40-week gestation newborn with a microphallus of 1.5 cm in length (normal values: 3.5 cm ± 0.4) and a terminal hypospadias with 3-ml testes located in the scrotal bag. He presented normal anthropometry. At 6 days of life, a hormonal study was carried out (**Table 1**), which showed total plasma testosterone values of 69 ng/dl. At 1 month of age, two 25-mg doses of intramuscular testosterone—separated by 2 weeks—were administered.

**Table 1:** Hormonal values, testosterone treatment, and penis length of our patient.

				hCG stimulation test^a^		
	6 d	1 mo	2 mo^b^	4 mo (pre-test)	4 mo + 3 w (post-test)	5 mo
Penis morphology/length	Microphallus, 1.5 cm		Microphallus, 2 cm	2.5 cm	3 cm	3.5 cm^b^
Testosterone treatment		25 mg × 2				25 mg × 2
LH	4.4 IU/L (2.7–7.6 IU/L, 1–30 d)^c^ (3.94 ± 3.19 IU/L, 7 d)^d^		4.8 U/L (0.12–4.8 IU/L, 1–3 mo)^c^	<0.12 U/L (0.05–1.1 IU/L)^c^	<0.12 U/L (0.05–1.1 IU/L)^c^	
FSH	3.8 U/L (0.6–7 U/L)		3.0 U/L	1.12 U/L	1.06 U/L	
**Testosterone**	69 ng/dl (33 ± 4 ng/dl, 4–7 d)^e^		**49 ng/dl** (196 ± 67 ng/dl, 2 to 3 mo)^e^	219.54 ng/dl (112 ± 50 ng/dl, 3 to 4 mo)^e^	111.1 ng/dl (78 ± 57 ng/dl, 4 to 5 mo)^e^	
**DHT**			**10 ng/dl** (6.5 ± 3.5 ng/ml)	**7 ng/dl**	**5 ng/dl**	
AMH			77.2 ng/ml (11.2–174.2 pg/dl)			
Inhibin B			192 pg/ml (87–243 pg/ml)			

In bold are low values.

DHT, dihydrotestosterone; AMH, anti-Müllerian hormone; d, days; w, weeks; mo, months; y, years.

a Six doses, 1,000 IU/day.

b After testosterone treatment.

c Reference serum levels (22).

d Reference serum levels (23).

e Basal plasma concentrations (mean ± SD) (24).

The patient was first seen at our hospital at 2 months of age. The length of the penis was 2 cm. To further characterize the patient, we calculated the external genital score (EGS). The EGS according to van der Straaten *et al.* (25) was 9.5 (undervirilization) and according to Ahmed and Rodie (26) was 8 (undervirilization). A new hormonal test that was performed (**Table 1**) showed low testosterone and DHT levels with inappropriately low values of LH for the levels of serum testosterone, normal thyroid and adrenal hormone profile, and normal prolactin and normal IGF-1 and IGFBP3 values. His karyotype was 46,XY. The hypothalamic–pituitary magnetic resonance was normal, and the ultrasound showed testes with homogeneous structure and of normal size. At the age of 4 months, a stimulation test with hCG at 1,000 IU/dose was performed (3 times/week for 2 weeks), which showed no significant increase in penis length or in the concentrations of testosterone (111.1 ng/dl) and DHT (0.05 ng/ml) (**Table 1**). Normal follicle-stimulating hormone (FSH), anti-Müllerian hormone, and inhibin B levels and 3-ml testicular volume ruled out gonadal dysgenesis.

**Figure 1:**
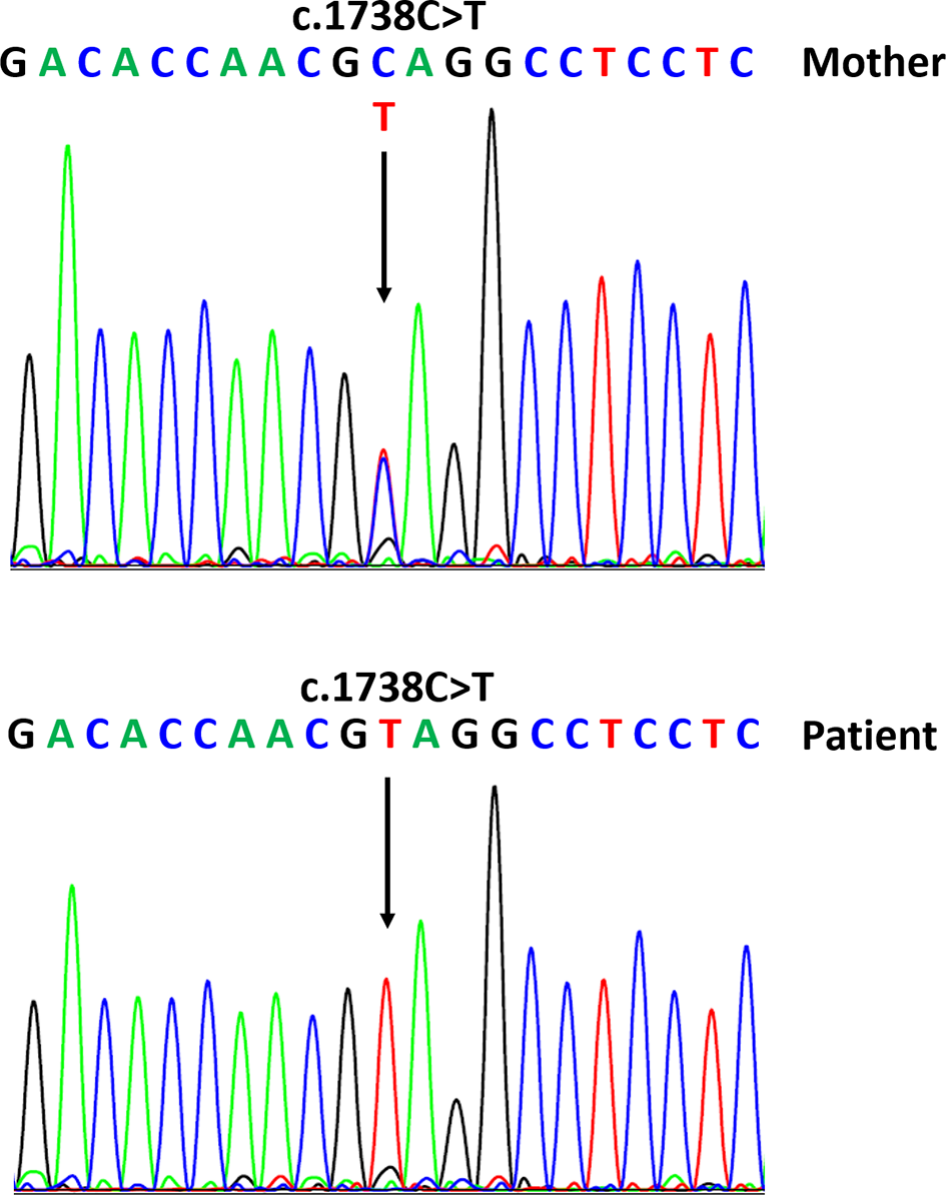
Sequences of the *MAMLD1* gene with the detected variant corresponding to our patient (hemizygous) and his mother (heterozygous).

To study both DSD and hypogonadotropic hypogonadism (HH), we performed two massive-sequencing gene panels: one including genes related to gonadal function (GeneRead DNA Library l Core Kit, Qiagen) and another including genes related to HH (Cell3 Target Custom Panel tier 2, NONACUS). The gonadal function gene panel included the genes *AR*, *CYP11A1*, *CYP17A1*, *HSD17B3*, *LHCGR*, *MAMLD1*, *NR0B1*, *NR5A1*, *SRD5A2*, *SRY*, *STAR*, and *WT1*. The HH panel included the genes *ANOS1*, *CDH7*, *FGF8*, *FGFR1*, *FSHB*, *GLI2*, *GNRH1*, *GNRHR*, *IL17RD*, *KISS1*, *KISS1R*, *KLB*, *LHB*, *MKRN3*, *NSMF*, *OTX2*, *PAX6*, *PROP1*, *SOX3*, *SOX10*, *TAC3*, *TACR3*, and *WDR11*. We identified a hemizygous nonsense variant in the *MAMLD1* gene (c.1738C>T:p.Gln580Ter) located in exon 3 (**Figure 1**). This variant was classified as pathogenic (ACMG fulfilled criteria PVS1, PM2, and PP3; ACMG classification; https://varsome.com) (27), and it has not been previously reported based on literature review and database [Human Genome Mutation Database (HGMD), Qiagen] search. No other genetic variants were detected.

At 5 months of age, treatment with intramuscular testosterone was started (50 mg in two doses every three weeks), showing a favorable response to treatment with an increase in penis length to 3.5 cm. The treatment was stopped when he was 6 months old.

## Discussion

We report a patient with hypospadias and microphallus with low testosterone, DHT, and LH levels due to a novel nonsense variant in the *MAMLD1* gene. The occurrence of hypospadias and microphallus is one of the manifestations of patients with *MAMLD1* variants (4, 14, 15, 19). Importantly, our patient showed a favorable response to treatment (intramuscular testosterone), with an increase in penis length of up to 3.5 cm.

Interestingly, our patient showed low testosterone and DHT levels and inappropriately normal LH levels in relation to testosterone at 2 months of age (**Table 1**), which, at first, let us to consider a case of partial hypogonadotropic hypogonadism with normal function of the FSH axis. At 2 months of age, after the first testosterone treatment, we expected a sensitization of the central axis and therefore an increase in LH. However, at 3 months of age, testosterone was already normal, whereas LH remained low. At 4 months, an hCG stimulation test was conducted: the basal testosterone values were normal before testing and decreased after, but LH remained low even post-hCG test. Furthermore, whereas it showed a decrease in testosterone levels, the DHT levels remained low through both minipuberty and hCG treatment. Our interpretation regarding these results is that the biosynthesis and LH axis were clearly affected in his first months after birth. Testosterone biosynthesis affectation would concur with a pathogenic variant in *MAMLD1*, whereas we suggest a possible implication of this gene in the LH axis. An alternative explanation for LH suppression may be that intramuscular testosterone (administered at 1 month of age) caused a decrease in LH. Regarding the decrease in testosterone levels after hCG stimulation, we believe that, although testosterone first increased, the decrease observed in the subsequent weeks may have been due to the child’s increasing age and physiological stage.

Although MAMLD1 has been shown to be involved in androgen biosynthesis (7), patients with *MAMLD1* variants mostly show normal gonadal function and normal testosterone levels (1, 4, 8, 12, 16, 18, 28). However, some patients do show low testosterone levels regardless of LH concentrations. Kalfa *et al.* have previously reported a *MAMLD1* patient with hypospadias with a significant reduction in plasma testosterone concentrations at 3 months of age (12, 15). This same patient also showed low concentrations of LH. Fukami *et al.* reported a patient with testosterone in the lower normal range and LH with normal values (low range) (1). Furthermore, Fujisawa reported a follow-up of this and other 2 patients (7–13 years of age) of the Fukami study (1) presenting with hypospadias and microphallus, which showed normal pre-hCG test testosterone levels but non-increased post-hCG. LH was normal at pre-test and elevated at post-test (8). Recently, Li and collaborators also reported some *MAMLD1* patients with low plasma testosterone levels, but in these cases, the LH concentrations ranged from low to normal (29). As far as we know, *MAMLD1* has not been related to LH axis regulation, yet other patients and ours present low LH levels. Regarding DHT, a few patients were reported, all with normal DHT levels at different ages in infancy (1, 13). However, this is the first report of low levels of DHT.

There are few reports on LH levels in patients with normal testosterone (at different ages), which are as follows: low-normal range LH (1), normal (low range) LH but increased LH after hCG stimulation (1, 8), and normal LH (high range) (15). Furthermore, a comparative study of testosterone and LH levels between *MAMLD1*-hypospadias patients and control individuals of the same age gave statistically significant differences only in the 4- to 8-year-old group and not at lower ages (28).

In our study, the testosterone, DHT, and gonadotropin concentrations during minipuberty (0 to 4 months of age) were measured. Both androgens and LH showed low serum levels and low pre- and post-hCG test levels (**Table 1**), with the exception of testosterone previous to the hCG test. We consider that the low testosterone and LH concentrations during this infant’s minipuberty stage and the non-significant increase in plasma concentrations of LH, testosterone, and DHT in the hCG test highlight the potential role of this gene in the biosynthesis of testosterone during the fetal stage and, specifically, in the minipuberty of the infant. Additionally, the LH values may suggest an implication of *MAMLD1* in the LH axis. Other groups have also reported values during minipuberty, and the results range from normal to low testosterone levels (1, 4, 12, 15, 29). Our group also reported a patient with hypospadias and microphallus with normal baseline testosterone (3 months) (4). These and our results expand the biochemical spectrum of patients with *MAMLD1* variants.

Up to now, there are over 40 *MAMLD1* reported variants (29–31) (HGMD database, Qiagen). The HGMD database reported to date 30 *MAMLD1* variants (including 2 long insertions) (HGMD Nov2021, Qiagen). This contrasts with the 41 single-nucleotide variants and small duplications/insertions in this gene reported in the latest and recent review (12). Forty were reported in 46,XY individuals and two in 46,XX female patients: one in homozygosity (12, 21) and one in heterozygosity (9). The homozygous variant (c.1514T>C:p.Val432Ala) in 46,XX has been confirmed to provide gain-of-function and has also been reported in other 46,XY male patients (4, 13, 14, 32). Moreover, some of the variants were reported in more than one family and report (12). It is worth noting that 13 variants reviewed by Miyado et al. were not included in HGMD (12) (HGMD database, Qiagen).

Regarding variants predicting a shorter protein product, nine *MAMLD1* nonsense variants have been reported (12). Our novel nonsense variant (c.1738C>T:p.Gln580Ter) *in silico* predicts a truncated protein and has not been detected in any control population database. It has been classified as pathogenic according to the ACMG criteria (https://varsome.com, November 2021) (27). Furthermore, there is strong evidence that nonsense *MAMLD1* variants are deleterious because all *MAMLD1* nonsense tested variants do show compromised transactivating activities (4, 6). All these evidences would concur with the production of a truncated protein or the absence of protein, maybe through nonsense-mediated decay mechanisms. Therefore, we consider that it could justify his genital and biochemical phenotype. Unfortunately, the MAMLD1 domains have only been described as amino acid-rich regions (6), and the Uniprot website describes the different domains as “disordered” (https://www.uniprot.org/uniprot/Q13495). To our knowledge, MAMLD1’s exact function and interaction properties still remain unclear. In particular, amino acid position 580 (p.Gln580Ter) is in the last part of the peptide sequence, after the domains described in Fukami’s work (6).

The *in vitro* and *in vivo* studies give evidence of the involvement of MAMLD1 in sex development, reproduction, and androgen production. Interestingly, *Mamld1*-knockdown *in vitro* assays in mouse Leydig tumor cells have shown a significant reduction of expression of different genes related to androgen biosynthesis, together with *Insl3*, related to testicular descent. This has led to low androgen levels in these cells (7). In contrast, normal testosterone concentrations have also been described in *Mamld1*-knockout fetal male mice (5), whereas these individuals have shown low mRNA levels of genes only expressed in the testes (*i*.*e*., *Star*, *Cyp11a1*, *Cyp17a1*, *Hsd3b1*, and *Insl3*) (5). Adult knockout mice present small testes with reduced seminiferous tubule size and proliferating germ cells but exhibit normal external genitalia and reproduce similarly to wild-type mice (5, 33). Moreover, *MAMLD1* is regulated by *NR5A1*/SF-1, which is involved in many processes of sex development and function (6), yet it has not been directly related to the regulation of the LH axis. There also seems to be a difference in its role between humans and mice and even in other species (12).

However, its specific function remains unclear and, lately, its role in sex development has become controversial. There are several reasons for this, including the fact that some *MAMLD1* variants have been detected in non-DSD individuals (control populations) (1, 14, 34, 35) and in patients with different DSD phenotypes (4). Furthermore, in some families, only part of the DSD individuals present a *MAMLD1* variant (1), and *in vitro* studies showed that several variants functioned similar to the wild type (4). Finally, *Mamld1*-knockout male mice have normal genitalia and reproduction (5, 33). These evidence have also led us to propose that *MAMLD1* may be linked to an oligogenic origin of the DSD of these *MAMLD1* patients (4, 9, 12, 31). The idea of various gene defects adding up to cause a specific disease/disorder has also been proposed in several endocrine/reproductive disorders such as hypogonadotropic hypogonadism (36), congenital hypothyroidism (37), and even in DSD related to *NR5A1* (38), thanks to high-throughput sequencing (39). Remarkably, our patient was suspected of partial hypogonadotropic hypogonadism due to his low LH levels, until we detected this *MAMLD1* variant. His biochemical characteristics also concur with some of the reported *MAMLD1* patients.

In line with the hypothesis of oligogenicity, we had previously performed a study searching for other variants in *MAMLD1* patients to explain the variability in DSD phenotypes (9). We detected additional variants in *WDR11* (40) and in the pituitary transcription factor *PROP1* (41) in one of the patients (hypospadias, microphallus, and small testes) and showed that PROP1 is part of the MAMLD1 network, connected to *MAMLD1* through NOTCH1/2 (9). Alternatively, the MAMLD1 defects may be directly linked to a partial affectation of the LH axis. There are reported evidences of MAMLD1 connections to pituitary hormone deficiency (9), signaling pathways (9), or gonadotropic cell proliferation and migration (specifically in gonadotroph pituitary adenomas) (42) that should be further explored. Taken together, our data may either reveal a new *MAMLD1* function related to LH axis regulation or strengthen the possibility of oligogenicity in our and similar *MAMLD1* patients.

## Concluding Remarks

Our patient presents a pathogenic variant in *MAMDL1* not previously described and that *in silico* predicts a truncated protein that could justify his genital phenotype. The existence of low testosterone, DHT, and LH concentrations during the infant’s minipuberty stage and the absence of a significant increase in plasma concentrations of testosterone and DHT in the hCG test highlight the potential role of this gene in the biosynthesis of testosterone during the fetal stage and minipuberty of the infant. Furthermore, LH values may suggest an implication of MAMLD1 in the LH axis or a possible oligogenicity. It is the first time that a decrease in DHT has been described in a patient with an abnormal *MAMLD1*. We have also demonstrated that intramuscular testosterone treatment is effective in these patients.

## Data Availability Statement

The datasets for this article are not publicly available due to concerns regarding participant/patient anonymity. Requests to access the datasets should be directed to the corresponding author.

## Ethics Statement

The studies involving human participants were reviewed and approved by the Comité Ético de Investigación Clínica y Comisión de Proyectos de Investigación del Hospital Universitari Vall d’Hebron (CEIC Hospital Universitari Vall d’Hebron). Written informed consent to participate in this study was provided by the participants’ legal guardian/next of kin.

## Author Contributions

DY, MC, CAR, and GC clinically and biochemically evaluated the patient. PFA performed the molecular analysis. DY and NCT contributed to the molecular evaluation and concept of the article. NCT drafted the article. All authors contributed to the article and approved the submitted version.

## Conflict of Interest

The authors declare that the research was conducted in the absence of any commercial or financial relationships that could be construed as a potential conflict of interest.

## Publisher’s Note

All claims expressed in this article are solely those of the authors and do not necessarily represent those of their affiliated organizations, or those of the publisher, the editors and the reviewers. Any product that may be evaluated in this article, or claim that may be made by its manufacturer, is not guaranteed or endorsed by the publisher.
